# A gene-to-patient approach uplifts novel disease gene discovery and identifies 18 putative novel disease genes

**DOI:** 10.1016/j.gim.2022.04.019

**Published:** 2022-05-09

**Authors:** Eleanor G. Seaby, Damian Smedley, Ana Lisa Taylor Tavares, Helen Brittain, Richard H. van Jaarsveld, Diana Baralle, Heidi L. Rehm, Anne O’Donnell-Luria, Sarah Ennis

**Affiliations:** 1 Human Development and Health, Faculty of Medicine, University of Southampton, Southampton, United Kingdom; 2 Program in Medical and Population Genetics, Broad institute of MIT and Harvard, Boston, MA; 3 Center for Genomic Medicine, Analytic and Translational Genetics Unit, Massachusetts General Hospital, Boston, MA; 4 Division of Genetics and Genomics, Boston Children’s Hospital, Boston, MA; 5 Genomics England, Dawson Hall, Charterhouse Square, London, EC1M 6BQ, United Kingdom; 6 Department of Genetics, University Medical Center Utrecht, Utrecht, The Netherlands

**Keywords:** Diagnostic uplift, Disease genes, Genome sequencing, Mendelian disease, Novel gene discovery

## Abstract

**Purpose:**

Exome and genome sequencing have drastically accelerated novel disease gene discoveries. However, discovery is still hindered by myriad variants of uncertain significance found in genes of undetermined biological function. This necessitates intensive functional experiments on genes of equal predicted causality, leading to a major bottleneck.

**Methods:**

We apply the loss-of-function observed/expected upper-bound fraction metric of intolerance to gene inactivation to curate a list of predicted haploinsufficient disease genes. Using data from the 100,000 Genomes Project, we adopt a gene-to-patient approach that matches de novo loss-of-function variants in constrained genes to patients with rare disease. Through large-scale aggregation of data, we reduce excess analytical noise currently hindering novel discoveries.

**Results:**

Results from 13,949 trios revealed 643 rare, de novo predicted loss-of-function events filtered from 1044 loss-of-function observed/expected upper-bound fraction–constrained genes. A total of 168 variants occurred within 126 genes without a known disease-gene relationship. Of these, 27 genes had >1 kindred affected, and for 18 of these genes, multiple kindreds had overlapping phenotypes. Two years after initial analysis, 11 of 18 (61%) of these genes have been independently published as novel disease gene discoveries.

**Conclusion:**

Using large cohorts and adopting gene-based approaches can rapidly and objectively accelerate dominantly inherited novel gene discovery by targeting the most appropriate genes for functional validation.

## Introduction

Next-generation sequencing has revolutionized rare disease diagnostics; more patients than ever are receiving a molecular diagnosis for their rare genetic disorders. This has been driven by the ever-increasing rise in novel disease gene discoveries, which is expanding the number of genes tested for in clinics.^1^ Making molecular genetic diagnoses is hugely important to patients and their families and can pave the way for therapeutic options, cascade testing, and family planning.^2^

However, most patients with rare diseases (up to 70% depending on clinical specialty) lack a definitive, molecular diagnosis.^3^ Clinical genetic testing often involves application of a gene panel either as the ordered test or by the analysis strategy applied to exome and genome sequencing.^4^ In the United Kingdom, the national genome sequencing program only reports on variants in a prespecified gene panel. Accredited clinical laboratories have no obligation to report on variants, including de novo variants outside of the panel applied.^5^ Yet, many patients harbor pathogenic variants not captured by a gene panel or in genes yet to be associated with disease. Indeed, approximately 50% of genes thought to cause disease through haploinsufficiency are yet to be associated with a clinical phenotype.^6,7^ Therefore, there is an unmet need for holistic and experimental approaches to identify novel disease genes and their associated phenotypes. These discoveries are critical for new genes to be added to diagnostic gene panels and for analytical approaches to uplift diagnostic rates.

### Current barriers to novel gene discovery

Novel disease gene discovery is a protracted process that requires identifying multiple, unrelated patients with variants in the same gene affected with similar phenotypes. These discoveries are then followed up with functional studies to provide evidence for gene causality.

Assessment of exome and genome data typically involves analysis of a small number of related individuals on a family-by-family basis. However, these analyses are time consuming and resource intensive, often requiring commercial software and cross-checking public databases. Each family member has 3 to 4 million variants in their genome and approximately 30,000 variants in their genes. Assessing every potential pathogenic variant is simply impossible.^3^ Although filtering techniques can restrict variant lists considerably, tens to thousands of variants of uncertain significance (VUS) typically remain with little to distinguish pathogenicity between them, particularly for genes of unknown function.^8^ It is not possible to investigate all potential candidate variants because this necessitates intensive functional experiments on variants of ostensibly equal predicted causality, which is proving to be a major bottleneck. Researchers are reluctant to invest in expensive studies without persuasive evidence that a given candidate warrants pursuing; however, identifying which variants should be prioritized is challenged by the paucity of knowledge into the function of most human genes. Therefore, these VUS end up as long lists of unreported variants present in a patient’s sequencing results that no one has time to resolve or investigate further. In many cases, these lists will contain the causal variant and thus represent missed opportunities for molecular diagnosis.

### The Matchmaker Exchange

One popular route to pursue candidate variants is through the Matchmaker Exchange (MME).^9^ MME has proven successful in building case series of patients with shared phenotypes involving the same gene, which are later taken to publication.^10^ However, this relies on knowing which gene candidates, of many, are best to submit to MME. Because of institutional restrictions on data sharing, it is not possible to query MME and return a list of genotypes and phenotypes for all submissions. Each match with another submitter requires electronic correspondence whereby both parties may choose to share variant- and genotype-specific data. Furthermore, there may be multiple matches per patient, making this method cumbersome and difficult to manage for large cohorts. Therefore, there are clear advantages to reducing the number of candidate variants for ongoing investigation.

### Gene constraint

Mutation is random, giving rise to new variants, most of which do not have a biological effect; however, some variants have greater consequences and may help us adapt and evolve, whereas others may be harmful and cause disease. Natural selection purges deleterious variation from human populations because fewer individuals with damaging variants survive and reproduce. However, in large population databases, such as the genome aggregation database (gnomAD), we still observe loss-of-function (LoF) variants because some genes are more tolerant than others to inactivation of one or even both gene alleles.^11^ We can exploit this principle to identify genes with fewer LoF variants observed in population data sets compared with random and expected variant rates, signifying genes most intolerant to LoF.^11,12^

Karczewski et al^11^ developed the loss-of-function observed/expected upper-bound fraction (LOEUF) score, which, for each gene in gnomAD v.2.1.1, compared the number of observed LoF variants in 125,748 individuals with the number expected. LOEUF places >19,000 genes along a continuous spectrum of intolerance to gene inactivation, whereby low scores, ie, the fewest predicted LoF (pLoF) variants observed compared with expectation, are the most intolerant to LoF. Indeed, genes in the first LOEUF decile (equivalent to a score < 0.2) have been validated as the most enriched for OMIM haploinsufficient disease genes and show the greatest biological essentiality.^11^ Yet, as of January 2021, 65% of genes in the lowest LOEUF decile are yet to have an OMIM disease association,^2^ leaving hundreds of undiscovered potential disease genes causing unrecognized phenotypes in patients.

Although statistical methods exist to identify potential novel disease genes using excess de novo mutation analysis, such as DeNovoWEST^13^ and DeNovolyzeR,^14^ these methods require huge cohorts of similar phenotypes, such as autism spectrum disorder. This study takes a nonstatistical approach across a more heterogenous cohort and aims to uplift novel disease gene discovery by targeting pLoF variants (with the greatest pathogenic potential) in genes whereby inactivation of a single copy of the gene is highly probable to cause dominant disease. We apply this method to the 100,000 Genomes Project, which has brought genome sequencing directly to patients with rare diseases in the United Kingdom.^15^ We move from a patient-to-gene approach to a gene-to-patient approach, whereby we are powered to identify and assign rare putative pathogenic variation in predicted disease genes to patients, cohort wide.

## Materials and Methods

### General methodological principle

**Figure 1:**
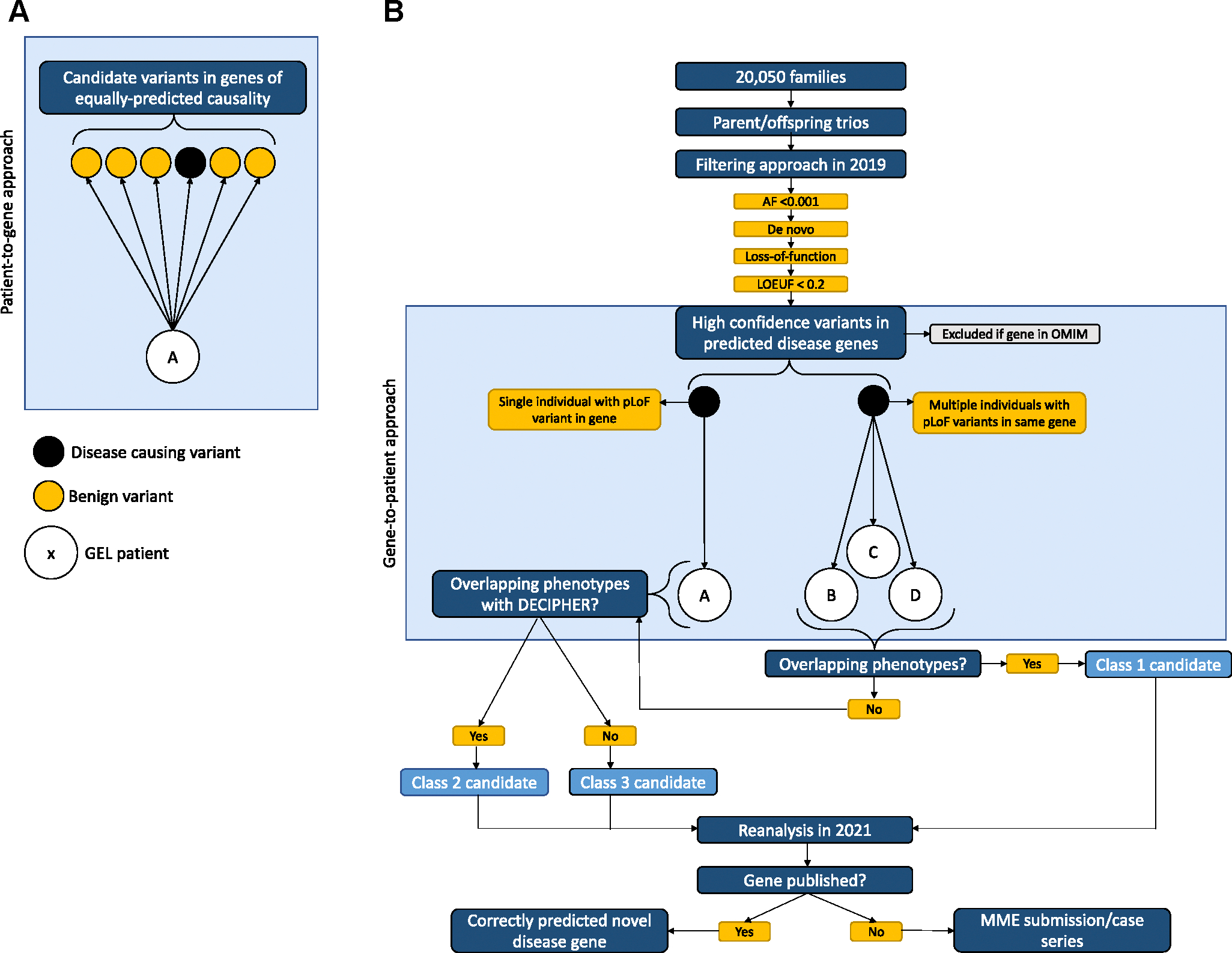
Method to uplift novel disease gene discovery. A. A typical patient-to-gene approach, whereby patient A’s exome or genome is analyzed and multiple candidates remain of similar predicted causality. B. A proposed gene-to-patient approach to identify novel disease genes that challenges the widely adopted diagnostic analytical paradigm of exome and genome sequencing. In this approach, a large-scale database is agnostically filtered for high-confidence, rare, de novo, pLoF variants in genes with an LOEUF < 0.2, and these variants are assigned to patients. Associated phenotypes are compared between patients with de novo pLoF variants in the same gene. AF, allele frequency; GEL, Genomics England; LOEUF, loss-of-function observed/expected upper-bound fraction; MME, Matchmaker Exchange; pLoF, predicted loss of function.

We propose an objective filtering strategy that can be applied at scale. We apply the LOEUF metric of intolerance to gene inactivation to define a list of predicted haploinsufficient disease genes. We select genes with an LOEUF score < 0.2 (first decile), which demonstrates the highest probability of representing autosomal dominant disease.^11^ By leveraging genomic and phenotypic data from rare disease trios in the 100,000 Genomes Project, we adopt an objective gene-to-patient approach that filters for rare de novo pLoF variants in LoF-constrained genes and matches these to rare disease patients. For this study, we exclude any variants in known OMIM disease genes (autosomal dominant or recessive) and focus only on novel disease genes. Where more than 1 patient with a de novo pLoF variant is found in the same gene, we call this a novel disease gene contender and then assess for phenotype overlap. This approach reduces analytical noise to focus on the most likely novel disease genes (Figure 1) and identifies suitable candidates for functional validation.

### Data access

Access to the secure Genomics England (GEL) research environment (RE) and high-performance cluster was obtained following information governance training and as a member of the Genomics England Clinical Interpretation Partnership: Quantitative methods, machine learning, and functional genomics and with approved project ID: RR359 - Translational genomics: Optimising novel gene discovery for 100,000 rare disease patients. This provided access, originally in 2019, to an aggregate vcf file of 20,050 rare disease families called using the Illumina Starling pipeline and passing quality control parameters as previously described.^5^ Most patients were children with neurodevelopmental disorders.^5^

Phenotype data for each patient were recorded by the referring clinician as a discrete list of Human Phenotype Ontology (HPO) terms.^16^ The number of HPO terms varied considerably between patients, with some individuals only having a single HPO term recorded. These data were stored within the RE in a LabKey data management system. The R LabKey package was used to extract HPO terms for each patient and merge these with genotype data.

### Code availability

Code generated for this project is specific to data securely held within the GEL RE. Only users with the necessary permission and governance training can access these data. Scripts are available to GEL users within the RE machine learning directory.

### Data filtering

Initial analysis was undertaken in October 2019. We selected full parent/offspring trios for de novo analysis, reducing the number of available families from 20,050 to 13,949. Bespoke scripts using bcftools^17^, VEP^18^, and Exomiser^19^ were developed to filter data. LOEUF scores were downloaded from gnomAD (http://gnomad.broadinstitute.org/downloads) and imported into the RE. We filtered out variants with an allele frequency (AF) > 0.001 across all gnomAD populations and retained only de novo pLoF variants (canonical splice site, frameshift, stop gain/nonsense, start loss, stop loss) on RefSeq transcripts called by VEP in genes with an LOEUF score < 0.2 to reflect genes with the greatest LoF constraint. To account for potential false positive pLoF calls, we applied LOFTEE v1.0 (https://github.com/konradjk/loftee), which removed low-confidence variants such as those in the last exon. Variants remaining after LOFTEE filtering were deemed high-confidence variants.

### Merging genotype data with additional datasets

High-confidence variants in putative disease genes that remained after the filtering approach in 2019 (AF < 0.001, de novo, pLoF, LOEUF < 0.2) were classified as either found in a known OMIM disease gene (already associated with disease) or in a non-OMIM disease gene (not yet associated with disease), achieved by querying the OMIM application program interface in October, 2019. All novel disease gene contenders were compared with 2 mouse databases, the International Mouse Phenotyping Consortium database and the Mouse Genome Informatics database.^20,21^

### Selecting high-priority novel disease gene candidates

High-confidence pLoF variants in novel disease gene contenders were selected as candidate pathogenic variants.

### Phenotype overlap

We assessed for phenotype overlap between unrelated patients who shared a candidate pLoF variant in the same gene. To do this, we computationally compared HPO terms (using their coded identification number) between individuals and considered a phenotype overlap to be when any single HPO matched exactly. Genes were prioritized as class 1 candidates if more than 1 unrelated patient harbored a candidate pathogenic variant in the same gene and there was a phenotype overlap (Table 1). These novel disease gene contenders were further curated against the literature to ascertain if there were existing publications implicating any of the genes as disease causing before being indexed in OMIM.

For novel disease gene contenders with only 1 pLoF variant in the cohort (ie, unique to 1 individual), we curated high-level phenotypes for each patient by manually upscaling their HPO terms to align with the terminology used in the publicly accessible Database of Genomic Variation and Phenotype in Humans Using Ensembl Resources (DECIPHER) database (http://deciphergenomics.org). For example, hydrocephalus was upscaled to “disorder of the nervous system” and atrial septal defect was coded as “disorder of the cardiovascular system”.^22^ We then compared high-level phenotypes of GEL patients with DECIPHER patients harboring de novo variants (pLoF or missense) in the same gene. We included do novo missense variants in DECIPHER to increase the number of genes with an associated phenotype for comparison. When high-level phenotypes matched, we classified these genes as class 2 candidates. In class 3 candidate genes, phenotypes did not match or no comparison was available (Table 1).

### Taking candidates forward

We requested permission to submit genes to GeneMatcher^23^ for class 1 genes by filling in request forms within the RE. We completed Clinician Contact Request forms for all class 1 candidates to obtain more detailed and current phenotype information from the patient’s referring clinician, in addition to obtaining consent to share genotypes and phenotypes and consent for publication with any matches made using the GeneMatcher node of MME. Where we successfully matched with international colleagues through MME and a case series was already underway, we worked with the patient’s clinician to include their patient in the existing case series. Where no case series were established, we initiated a new interest group to lead on collecting phenotype data from collaborators and started functional experiments in Xenopus on novel disease gene contenders.

### Validation of method

**Figure 2:**
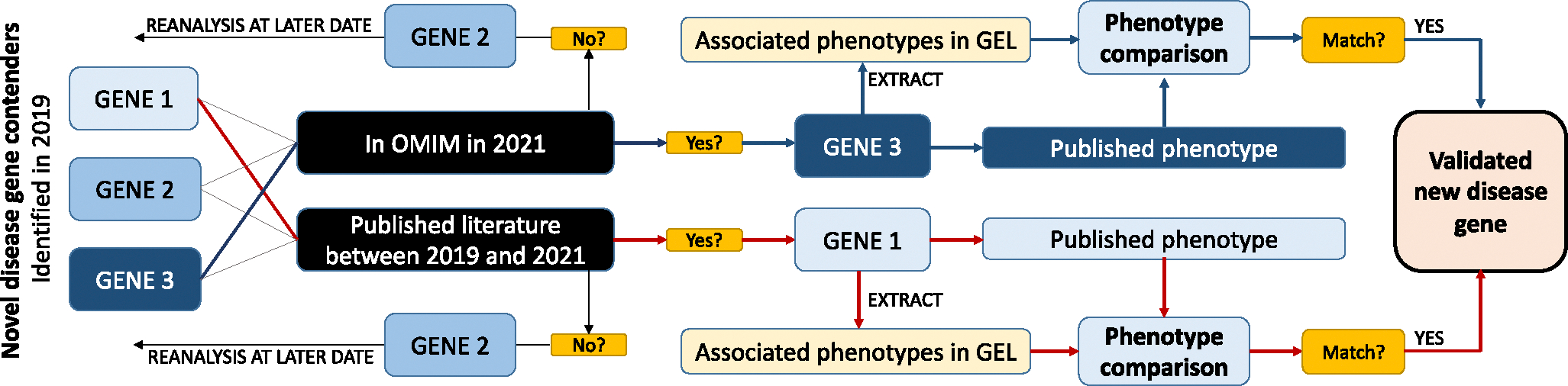
Workflow applied for the validation of novel disease gene contenders. Workflow applied to validate novel disease gene contenders as new disease genes. The red path shows the pathway for gene 1, which was identified in 2019 and published in the literature between 2019 and 2021. Associated phenotypes of GEL patients with pLoF in gene 1 were compared with the published phenotype and matched, validating gene 1 as a correctly predicted new disease gene. The blue path shows the pathway for gene 2; this gene was compared with OMIM and the literature and was not yet published/in OMIM and will be recompared at a later stage. Gene 3 (thin gray line) was present in OMIM by 2021 and associated phenotypes in GEL patients with pLoF variants in gene 3 overlapped with the published literature, validating this gene as a correctly predicted new disease gene. GEL, Genomics England; pLoF, predicted loss of function.

To validate whether we could correctly predict novel disease genes, we compared our novel disease gene contenders in 2019 against an updated list of dominant OMIM disease genes from 2021, in addition to literature published between 2019 and 2021. If one of our predicted novel disease gene contenders from 2019 was added to OMIM or was published as a disease gene between 2019 and 2021, we manually compared the HPO terms of GEL patients with the clinical phenotypes reported in the literature and/or OMIM to assess concordance (Figure 2). We considered our method as having correctly predicted a disease gene when any of the patients in GEL had significant overlapping features with the clinical presentation published for variants in the same gene and the GEL variant met, at minimum, likely pathogenic status by American College of Medical Genetics and Genomics/Association for Molecular Pathology guidelines.^24,25^ We further assessed whether any alternative diagnoses were made by National Health Service–accredited genetics laboratories between 2019 and 2021.

## Results

**Figure 3:**
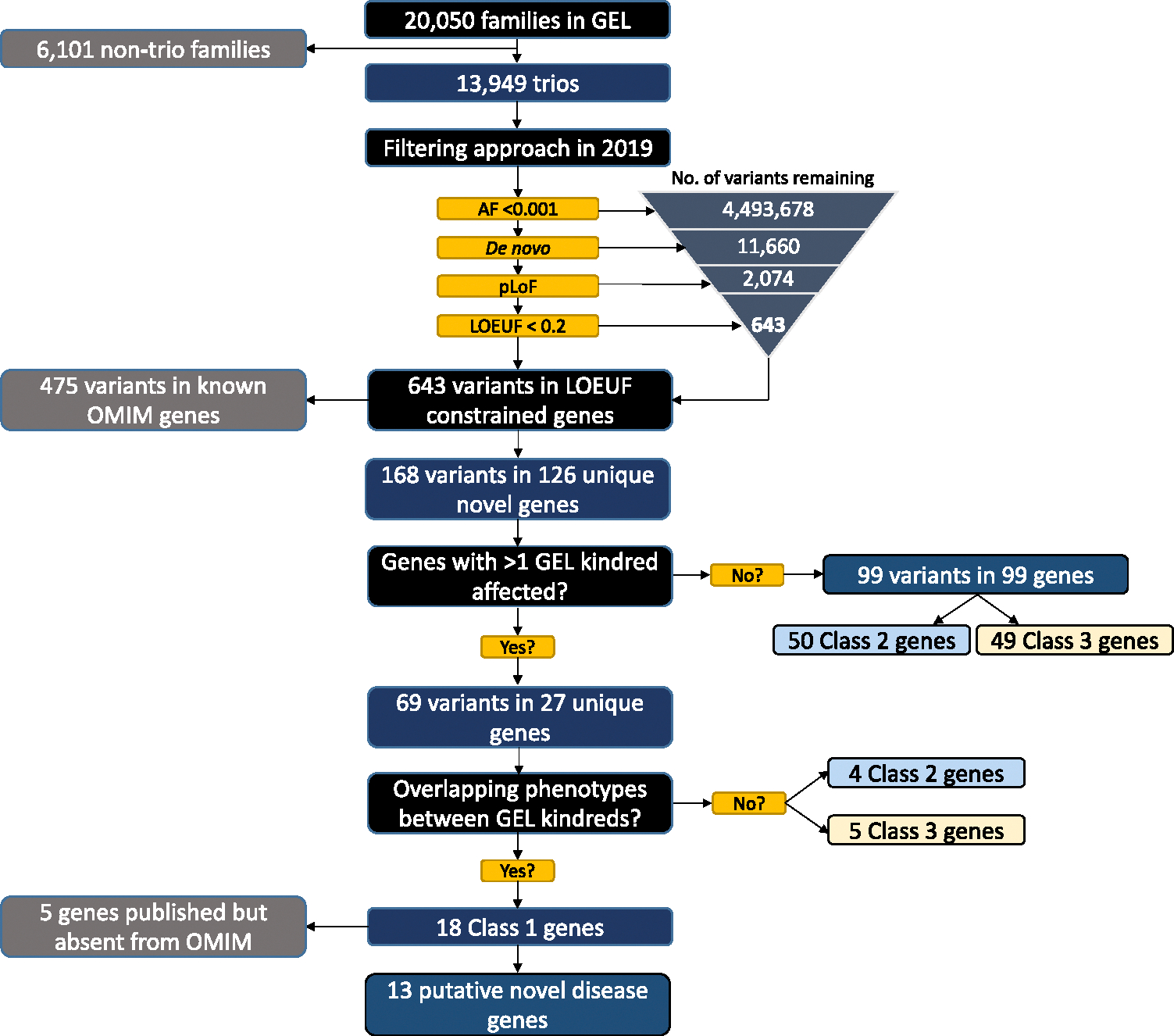
Summary of class 1, 2, and 3 results. Summary of gene discovery results after filtering variants from GEL families. GEL, Genomics England.

Data from the 100,000 Genomes Project (13,949 trios, involving 41,847 individuals) revealed 643 rare (AF < 0.001) de novo pLoF events filtered in 1044 pLoF-constrained genes (Figure 3). A total of 475 variants were in 148 known OMIM genes (as of October, 2019) and 168 were in novel disease gene contenders (involving 126 unique genes). Of these, 27 genes had more than one GEL kindred affected and 18 had overlapping phenotypes, meeting class 1 criteria (Table 1). Of these class 1 genes, 5 were absent from OMIM but had been published in the literature (Supplemental Table 1). Six more of these genes have since been published as disease-causing genes with matching phenotypes to our GEL probands (Table 2).

Nine genes had more than one GEL kindred affected but the phenotypes between patients were nonoverlapping, meaning that there were no exact matches of HPO terms between patients; 4 genes met class 2 criteria with high-level phenotypes overlapping with DECIPHER entries, and 5 genes met class 3 criteria (Supplemental Table 1).

A total of 99 variants in 99 unique genes were identified in 98 individuals (Supplemental Table 2). Of these, 50 genes were classified as class 2 candidates, meaning that their high-level phenotypes overlapped with individuals in DECIPHER harboring de novo pLoF or missense variants in the same gene. A total of 49 genes were class 3, meaning no patients within GEL or DECIPHER had matching phenotypes involving the same gene.

### Investigating and validating putative disease genes

Between 2019 and 2021, 23 of 126 (18%) of our novel disease gene contenders were published by independent groups. Class 1 candidates were the highest predictors of disease genes, with 11 of 18 (61%) having been functionally validated and published, confirming their status as new disease genes.

Of the class 2 and class 3 genes occurring in unique individuals, 2 of 50 (4%) and 10 of 49 (20%), respectively, have been published with evidence of causality. Of the remaining 7 class 1 genes yet to be validated, case series/and or functional experiments are underway. By 2021, 15 patients had likely pathogenic or pathogenic variants independently identified in alternative known disease genes by GEL diagnostic laboratories. In total, we identified 126 novel disease gene contenders.

## Discussion

We rapidly applied an objective filtering strategy across a large cohort and identified 18 high-confidence putative novel disease genes, of which 11 (61%) have since been validated through functional experiments and confirmed as disease causing. Additionally, we identified a further 108 novel disease gene contenders.

In total, 23 of 126 (18%) of the genes identified in our study have been validated as disease causing, and diagnoses are being returned to patients who would otherwise have a negative genome report. This was achieved by a targeted gene-to-patient approach applied to the 100,000 Genomes Project with the power to detect very rare pLoF variation in genes most intolerant to LoF. However, only in time will we determine the full specificity and sensitivity of this approach.

### Class 1 genes and internal matches in GEL

Since initial analysis, 11 of 18 (61%) class 1 genes (Table 1) have undergone functional validation and been published by independent groups confirming their status as novel disease gene discoveries, and we anticipate this number to increase over time. Class 1 genes outperformed classes 2 and 3 (Fisher’s exact test < 0.0001) likely because of the greater specificity and granularity of phenotypes available for internal matching within GEL.

There were 9 genes where unrelated patients in GEL had pLoF variants in the same gene, yet no patient shared the same HPO terms. However, 3 of these genes have since been published, and the published phenotypes overlap with the GEL patients (Supplemental Table 1). This may be explained by variability in HPO terms reported in GEL; some patients had many HPO terms recorded, yet others had only 1 or 2. In Table 2, patients with pLoF variants in *SPEN* and *TANC2* only overlapped by 1 HPO term (intellectual disability). Yet, when the disease phenotype was further delineated in published case series for both genes, many more features observed in the GEL patients were consistent with the reported phenotypic spectrum. This highlights the need for longitudinal and deep phenotyping data in automated gene discovery studies.

Owing to the automated process of exact HPO term matching between GEL patients, we potentially missed overlapping phenotypes recorded with subtly different nomenclature, eg, one patient with intellectual disability (HP: 0001249) would not match another patient with mild intellectual disability (HP: 0001256).

### Class 2 and 3 genes

More class 3 genes (10/50, 20%) were published as novel causal genes by 2021 than class 2 genes (2/49, 4%; Fisher’s exact test = 0.027). This may be due to small sample sizes but could reflect a weakness in class 2 and 3 classification (Table 1). Comparing high-level phenotypes is potentially problematic because it lacks the granularity required to assess clinical overlap. Furthermore, we compared high-level phenotypes of GEL patients with patients in DECIPHER harboring de novo missense variants, which are considerably more common and less likely to be pathogenic, increasing the possibility of false disease-phenotype associations. Additionally, it is possible that some patients in our cohort were also in DECIPHER; however, because of data anonymity, this could not be verified.

### Lessons learned

Class 2 and class 3 genes may be better assessed through MME. Sharing more detailed phenotype data would provide the granularity to assess true clinical overlap. In GEL, this step involved contacting the patient’s clinician for permission to share data with matches through MME, and this process was not always successful. Because MME involves manual correspondence between peers, this cannot be easily automated, highlighting the advantages of internal phenotype matching within the same cohort. We are fully utilizing MME for novel gene candidates; however, presenting these results is outside of the scope of this manuscript.

### Novel gene discovery remains time consuming

Although our method is rapid at identifying highly promising novel candidate genes, there remain persistent time requirements to validate any results through case series and functional experiments; however, the strength of the method is in rapidly identifying which VUS to pursue and therefore shortening the process of discovery. We have identified 7 class 1 genes, for which we are accruing case series on 6 genes and have started functional studies in 4 genes (Table 2). We believe our method provides the opportunity to identify the most salient candidates for follow-on studies, meaning that many patients will have their most damaging VUS investigated when, typically, no candidates would have been pursued.

### Considerations and limitations

#### De novo analysis applied to predominantly neurodevelopmental phenotypes

We used trios for de novo analysis, meaning that families without trio data were excluded. However, with plans to sequence 5 million more genomes in the United Kingdom, we believe our method will prove increasingly more effective. Furthermore, we plan to refine our analysis to query all affected individuals in GEL with pLoF variants in our novel disease gene contenders, even if segregation data is unavailable.

The 100,000 Genomes Project is enriched for patients with rare neurodevelopmental disorders, and therefore we risk comparing patient phenotypes within a cohort enriched for similar phenotypes. We were cautious of defining phenotype overlap as any 2 patients exactly matching on 1 HPO term; however, because of the variability in number of HPO terms reported in GEL, this maximized sensitivity of class 1 genes and enabled us to correctly predict *SPEN* and *TANC2* as novel disease genes.

#### Statistical rigor

The large number of neurodevelopmental disorders in the dataset caused by many heterogenous genes precludes the reliability of statistical methods to confirm/refute novel disease gene contenders, although with ongoing genome sequencing in the United Kingdom, this will likely be overcome. Further, we specifically focused on pLoF variants only, meaning that we are not powered even with 3 de novo variants per gene (the maximum we observed for class 1 candidates) to reach statistical significance using a case/control Fisher’s test and multiple test correction.^14^ Instead, we rely on the established approach of identifying overlapping phenotypes to further prioritize the best candidates for functional validation.

#### Minor allele frequency

We used a liberal AF of < 0.001, yet the highest variant frequency we observed was 0.0002. The presence of these rare de novo variants within gnomAD could represent recurrent de novo variation.^32^ Although a more restrictive AF would increase confidence of pathogenicity, pathogenic disease variants can be present in population databases owing to incomplete penetrance, effects of cis-regulatory variation, and adult-onset disease.^33^ Nevertheless, our cohort is likely depleted for adult-onset diseases, because early-onset conditions are more likely to have complete trios.

#### Prioritizing haploinsufficiency

Our method is enriched for haploinsufficient disease genes, and we did not prioritize biallelic observations in our analysis.^11^ Using an LOEUF score < 0.2 (top decile) enabled us to select the genes most highly constrained for LoF, although the expectation was that these would be associated with dominant inheritance, meaning that our approach is not enriched for autosomal recessive novel gene discovery. Several genes in the top decile may be embryonically lethal, although we do not expect to observe these in our cohort. With higher LOEUF thresholds, it is likely that further haploinsufficient disease genes and even more recessive disease genes will be found but at the expense of increased noise.^11^

#### Classification of pLoF variants

We included start loss and stop loss within the category of LoF variants; however, these variants often do not constitute true LoF and show selection signatures more similar to missense variants.^34^ We only observed 6 start/stop loss variants, and therefore potential misclassification of these variants is not expected to have substantively affected our analysis. Our current analysis strategy also misses other LoF variants, eg, untranslated region variants, extended splice site, and structural variants. Research into these potential LoF-disrupting variants using tools such as UTRannotator^35^ and spliceAI^36^ may further expand the disease gene candidate list.

#### False positive pLoF variants

Not all pLoF variants truly cause LoF; many are enriched for technical, rescue, and affect errors.^11,33^ Although in silico tools can identify some of these errors, manual curation is the most effective method to identify potential false positives.^33^ However, this process is extremely time consuming and not yet standardized; therefore, there is risk that we included false positive LoF variants in our analysis.^33^ We expect these false positives are more likely to be variants with higher allele frequencies in gnomAD or in class 2 and 3 genes whereby detailed phenotype data cannot be assessed for overlap. Indeed, 15 of our pLoF variants in class 2 and 3 genes were in individuals who had an alternative pathogenic variant (Supplemental Table 2). Although this does not rule out the potential for a second diagnosis, which occurs up to 5% of the time,^6^ it does raise the possibility of a variant without functional effect.

## Conclusion

Using a large cohort and adopting a highly efficient gene-based approach can accelerate novel gene discovery and target the most appropriate variants and genes for functional validation. This can uplift diagnostic rates and add new disease genes to clinical gene panels.

As rare disease cohorts continue to increase, there is increasing demand to automate analyses and reduce the burden of variants requiring analysis by clinical scientists. With increasing study sizes, our method should be better powered to detect rare pathogenic variation shared across individuals but necessitates real-time comparison to previously generated large datasets if the approach is to be used in routine diagnostics. Assessing phenotype overlap is an important step in our method, and with drives toward data sharing, there is opportunity to securely access data and apply automated phenotype matching within and across cohorts using trusted REs, such as the National Human Genome Research Institute’s Genomic Data Science Analysis, Visualisation, and Informatics Lab space.^37^

We anticipate that our method can be applied by other researchers to their own cohorts; however, we emphasize the importance of trio analyses and encourage prudence when determining what constitutes LoF. We demonstrate that gene-based approaches can successfully identify novel disease genes, and with larger rare disease cohorts, it is hoped that more discoveries will be identified for the benefit of patients, their families, and the wider scientific community.

## Supplementary Material

1

## Figures and Tables

**Table 1:** Classification of novel disease gene contenders

Gene Class	Classification Rule

Class 1	pLoF variants identified in the same gene in 2 or more unrelated kindred in GEL and at least 1 HPO term exactly matched between affected individuals.
Class 2	A pLoF variant identified in a gene in 1 affected individual in GEL, whereby at least 1 high-level phenotype exactly overlapped between the GEL patient and an individual in DECIPHER with a de novo pLoF or missense variant in the same gene.
Class 3	No overlap in HPO terms between patients in GEL with pLoF variants in the same gene, or no overlap in high-level phenotypes between GEL patients and affected patients in DECIPHER with de novo pLoF or missense variants in the same gene (or no available phenotype in DECIPHER for comparison).

*DECIPHER,* Database of Genomic Variation and Phenotype in Humans Using Ensembl Resources; *GEL,* Genomics England; *HPO,* Human Phenotype Ontology;pLoF, predicted loss-of-function.

**Table 2:** Thirteen putative novel disease genes

Gene	Consequence	MaxFreq	Shared HPO Terms Across Patients in GEL	Overlapping Features Between GEL Patients and Published Literature	Publication Status (June 2021)

*HDLBP*	Frameshift	Absent	Macrocephaly, intellectual disability, global developmental delay, delayed speech and language development, delayed fine and gross motor development, autism	N/A	Case series and functional studies underway
	Frameshift	Absent			
	Start lost	Absent			
*RIF1*	Frameshift	Absent	Delayed speech and language development, global developmental delay, delayed gross motor development, intellectual disability	N/A	Case series and functional studies underway
	Frameshift	Absent			
*DDX17*	Frameshift	Absent	Horizontal nystagmus, global developmental delay, skeletal abnormalities	N/A	Case series and functional studies underway
	Splice acceptor	Absent			
*TAF4*	Frameshift	Absent	Seizures, spasticity, brain atrophy, cerebellar signs	N/A	Manuscript in preparation
	Stop gained	Absent			
	Splice donor	0.00008			
*CLASP1*	Stop gained	0.00020	Delayed speech and language, global developmental delay, delayed gross motor development, intellectual disability	N/A	Case series underway
	Stop lost	Absent			
*ANKRD12*	Splice acceptor	Absent	Intellectual disability, global developmental delay	N/A	Case series and functional studies underway
	Frameshift	0.00001			
	Frameshift	Absent			
*CASZ1*	Frameshift	Absent	Intellectual disability	N/A	Case series underway
	Frameshift	Absent			
*ZNF292* ^a^	Frameshift	0.00001	Global developmental delay, facial shape abnormalities, intellectual disability	Intellectual disability, global developmental delay, delayed speech, microcephaly, skeletal abnormalities, seizures, dysmorphic features, abnormal face shape	Mirzaa et al^26^
	Frameshift	Absent			
*SETD1A* ^a^	Stop gained	Absent	Global developmental delay, intellectual disability	Delayed speech and language development, intellectual disability, seizures, global developmental delay, dysmorphic facial features, hypotonia	Yu et al^27^
	Frameshift	Absent			
*ANKRD17* ^a^	Stop gained	Absent	Delayed speech and language development, delayed gross motor development, intellectual disability	Intellectual disability, delayed speech and language development, dysmorphic features	Chopra et al^28^
	Frameshift	Absent			
*USP7* ^a^	Stop gained	Absent	Global developmental delay and abnormal facial shape	Intellectual disability, seizures, hypotonia, global developmental delay, facial shape deformation, feeding difficulties	Fountain et al^29^
	Stop gained	0.00007			
*TANC2* ^a^	Stop gained	Absent	Intellectual disability	Intellectual disability, global developmental delay, behavioral abnormalities, autism, impaired speech development, seizures, delayed motor development	Guo et al^30^
	Frameshift	Absent			
*SPEN* ^a^	Stop gained	Absent	Intellectual disability	Developmental delay/intellectual disability, autism spectrum disorder, behavioral abnormalities, dysmorphic features, obesity/increased BMI	Radio et al^31^
	Stop gained	Absent			

A table of 13 putative novel disease genes identified from analysis in 2019. Shared phenotypes between patients involving pLoF variants in the same gene are listed.

*BMI,* body mass index; *GEL*, Genomics England; *HPO*, Human Phenotype Ontology; *MaxFreq*, maximum allele frequency in gnomAD v2.1.1 and 1000 Genomes phase 3 data; *N/A*, not available for comparison; *pLoF*, predicted loss of function.

a Candidates that have been published as of June 2021. For these, shared phenotypes between patients in GEL and patients included in publications by 2021 are recorded.

## Data Availability

The anonymized phenotype and genotype data that support the findings of this study are only available as a registered Genomics England Clinical Interpretation Partnership member in the Genomics England Research Environment, but restrictions apply to the availability of these data because of data protection and are not publicly available. Information regarding how to apply for data access is available at the following URL: https://www.genomicsengland.co.uk/about-gecip/for-gecip-members/data-and-data-access/. No individual’s variant or phenotype data can be shared or published because of data sharing restrictions. All data shared in this manuscript were approved for export by Genomics England.

## Members of the Genomics England Research Consortium

J.C. Ambrose, P. Arumugam, R. Bevers, M. Bleda, F. Boardman-Pretty, C.R. Boustred, H. Brittain, M.J. Caulfield, G.C. Chan, T. Fowler, A. Giess, A. Hamblin, S. Henderson, T.J.P. Hubbard, R. Jackson, L.J. Jones, D. Kasperaviciute, M. Kayikci, A. Kousathanas, L. Lahnstein, S.E.A. Leigh, I.U.S. Leong, F.J. Lopez, F. Maleady-Crowe, M. McEntagart, F. Minneci, L. Moutsianas, M. Mueller, N. Murugaesu, A.C. Need, P. O’Donovan, C.A. Odhams, C. Patch, D. Perez-Gil, M.B. Pereira, J. Pullinger, T. Rahim, A. Rendon, T. Rogers, K. Savage, K. Sawant, R.H. Scott, A. Siddiq, A. Sieghart, S.C. Smith, A. Sosinsky, A. Stuckey, M. Tanguy, A.L. Taylor Tavares, E.R.A. Thomas, S.R. Thompson, A. Tucci, M.J. Welland, E. Williams, K. Witkowska, S.M. Wood.

## References

[1] Bamshad MJ, Nickerson DA, Chong JX. Mendelian gene discovery: fast and furious with no end in sight. Am J Hum Genet. 2019;105(3):448–455. 10.1016/j.ajhg.2019.07.011. 31491408 PMC6731362

[2] Seaby EG, Rehm HL, O’Donnell-Luria A. Strategies to uplift novel Mendelian gene discovery for improved clinical outcomes. Front Genet. 2021;12:674295. 10.3389/fgene.2021.674295. 34220947 PMC8248347

[3] Seaby EG, Ennis S. Challenges in the diagnosis and discovery of rare genetic disorders using contemporary sequencing technologies. Brief Funct Genomics. 2020;19(4):243–258. 10.1093/bfgp/elaa009. 32393978

[4] Martin AR, Williams E, Foulger RE, PanelApp crowdsources expert knowledge to establish consensus diagnostic gene panels. Nat Genet. 2019;51(11):1560–1565. 10.1038/s41588-019-0528-2. 31676867

[5] 100,000 Genomes Project Pilot Investigators, Smedley D, Smith KR, 100,000 Genomes pilot on rare-disease diagnosis in health care—preliminary report. N Engl J Med. 2021;385(20):1868–1880. 10.1056/NEJMoa2035790. 34758253 PMC7613219

[6] Posey JE, O’Donnell-Luria AH, Chong JX, Insights into genetics, human biology and disease gleaned from family based genomic studies. Genet Med. 2019;21(4):798–812. 10.1038/s41436-018-0408-7. 30655598 PMC6691975

[7] Chong JX, Buckingham KJ, Jhangiani SN, The genetic basis of Mendelian phenotypes: discoveries, challenges, and opportunities. Am J Hum Genet. 2015;97(2):199–215. 10.1016/j.ajhg.2015.06.009. 26166479 PMC4573249

[8] Smedley D, Schubach M, Jacobsen JOB, A whole-genome analysis framework for effective identification of pathogenic regulatory variants in Mendelian disease. Am J Hum Genet. 2016;99(3):595–606. 10.1016/j.ajhg.2016.07.005. 27569544 PMC5011059

[9] Philippakis AA, Azzariti DR, Beltran S, The Matchmaker Exchange: a platform for rare disease gene discovery. Hum Mutat. 2015;36(10):915–921. 10.1002/humu.22858. 26295439 PMC4610002

[10] Azzariti DR, Hamosh A. Genomic data sharing for novel Mendelian disease gene discovery: the Matchmaker Exchange. Annu Rev Genomics Hum Genet. 2020;21:305–326. 10.1146/annurevgenom-083118-014915. 32339034

[11] Karczewski KJ, Francioli LC, Tiao G, The mutational constraint spectrum quantified from variation in 141,456 humans. Nature. 2020;581(7809):434–443. Published correction appears in Nature. 2021;590(7846):E53. Published correction appears in Nature. 2021;597(7874):E3–E4. 10.1038/s41586-020-2308-7 32461654 PMC7334197

[12] Collins RL, Brand H, Karczewski KJ, A structural variation reference for medical and population genetics. Nature. 2020;581(7809):444–451. Published correction appears in Nature. 2021;590(7846):E55. 10.1038/s41586-020-2287-8 32461652 PMC7334194

[13] Kaplanis J, Samocha KE, Wiel L, Evidence for 28 genetic disorders discovered by combining healthcare and research data. Nature. 2020;586(7831):757–762. 10.1038/s41586-020-2832-5. 33057194 PMC7116826

[14] Samocha KE, Robinson EB, Sanders SJ, A framework for the interpretation of de novo mutation in human disease. Nat Genet. 2014;46(9):944–950. 10.1038/ng.3050. 25086666 PMC4222185

[15] POSTnote. The 100,000 Genomes Project. Parliamentary Office of Science and Technology; 2015. Accessed October 2019. https://researchbriefings.files.parliament.uk/documents/POST-PN-0504/POSTPN-0504.pdf.

[16] Robinson PN, Köhler S, Bauer S, Seelow D, Horn D, Mundlos S. The Human Phenotype Ontology: a tool for annotating and analyzing human hereditary disease. Am J Hum Genet. 2008;83(5):610–615. 10.1016/j.ajhg.2008.09.017. 18950739 PMC2668030

[17] Li H, Handsaker B, Wysoker A, The Sequence Alignment/Map format and SAMtools. Bioinformatics. 2009;25(16):2078–2079. 10.1093/bioinformatics/btp352. 19505943 PMC2723002

[18] McLaren W, Gil L, Hunt SE, The Ensembl variant effect predictor. Genome Biol. 2016;17(1):122. 10.1186/s13059-016-0974-4. 27268795 PMC4893825

[19] Smedley D, Jacobsen JOB, Jäger M, Next-generation diagnostics and disease-gene discovery with the Exomiser. Nat Protoc. 2015;10(12):2004–2015. 10.1038/nprot.2015.124. 26562621 PMC5467691

[20] Muñoz-Fuentes V, Cacheiro P, Meehan TF, The International Mouse Phenotyping Consortium (IMPC): a functional catalogue of the mammalian genome that informs conservation. Conserv Genet. 2018;19(4):995–1005. 10.1007/s10592-018-1072-9. 30100824 PMC6061128

[21] Bult CJ, Blake JA, Smith CL, Kadin JA, Richardson JE. Mouse Genome Database Group. Mouse Genome Database (MGD) 2019. Nucleic Acids Res. 2019;47(D1):D801–D806. 10.1093/nar/gky1056. 30407599 PMC6323923

[22] Firth HV, Wright CF, DDD Study. The Deciphering Developmental Disorders (DDD) study. Dev Med Child Neurol. 2011;53(8):702–703. 10.1111/j.1469-8749.2011.04032.x. 21679367

[23] Sobreira N, Schiettecatte F, Valle D, Hamosh A. GeneMatcher: a matching tool for connecting investigators with an interest in the same gene. Hum Mutat. 2015;36(10):928–930. 10.1002/humu.22844. 26220891 PMC4833888

[24] Abou Tayoun AN, Pesaran T, DiStefano MT, Recommendations for interpreting the loss of function PVS1 ACMG/AMP variant criterion. Hum Mutat. 2018;39(11):1517–1524. 10.1002/humu.23626. 30192042 PMC6185798

[25] Richards S, Aziz N, Bale S, Standards and guidelines for the interpretation of sequence variants: a joint consensus recommendation of the American College of Medical Genetics and Genomics and the Association for Molecular Pathology. Genet Med. 2015;17(5):405–424. 10.1038/gim.2015.30. 25741868 PMC4544753

[26] Mirzaa GM, Chong JX, Piton A, De novo and inherited variants in ZNF292 underlie a neurodevelopmental disorder with features of autism spectrum disorder. Genet Med. 2020;22(3):538–546. 10.1038/s41436-019-0693-9. 31723249 PMC7060121

[27] Yu X, Yang L, Li J, De novo and inherited SETD1A variants in early-onset epilepsy. Neurosci Bull. 2019;35(6):1045–1057. 10.1007/s12264-019-00400-w. 31197650 PMC6864154

[28] Chopra M, McEntagart M, Clayton-Smith J, Heterozygous ANKRD17 loss-of-function variants cause a syndrome with intellectual disability, speech delay, and dysmorphism. Am J Hum Genet. 2021;108(6):1138–1150. 10.1016/j.ajhg.2021.04.007. 33909992 PMC8206162

[29] Fountain MD, Oleson DS, Rech ME, Pathogenic variants in USP7 cause a neurodevelopmental disorder with speech delays, altered behavior, and neurologic anomalies. Genet Med. 2019;21(8):1797–1807. 10.1038/s41436-019-0433-1. 30679821 PMC6752677

[30] Guo H, Bettella E, Marcogliese PC, Disruptive mutations in TANC2 define a neurodevelopmental syndrome associated with psychiatric disorders. Nat Commun. 2019;10(1):4679. 10.1038/s41467-019-12435-8. 31616000 PMC6794285

[31] Radio FC, Pang K, Ciolfi A, SPEN haploinsufficiency causes a neurodevelopmental disorder overlapping proximal 1p36 deletion syndrome with an episignature of X chromosomes in females. Am J Hum Genet. 2021;108(3):502–516. 10.1016/j.ajhg.2021.01.015. 33596411 PMC8008487

[32] Wilfert AB, Sulovari A, Turner TN, Coe BP, Eichler EE. Recurrent de novo mutations in neurodevelopmental disorders: properties and clinical implications. Genome Med. 2017;9(1):101. 10.1186/s13073-017-0498-x. 29179772 PMC5704398

[33] Gudmundsson S, Karczewski KJ, Francioli LC, Addendum: the mutational constraint spectrum quantified from variation in 141,456 humans. Nature. 2021;597(7874):E3–E4. 10.1038/s41586-021-03758-y. 34373650 PMC8410591

[34] Lek M, Karczewski KJ, Minikel EV, Analysis of protein-coding genetic variation in 60,706 humans. Nature. 2016;536(7616):285–291. 10.1038/nature19057. 27535533 PMC5018207

[35] Zhang X, Wakeling M, Ware J, Whiffin N. Annotating high-impact 5′ untranslated region variants with the UTRannotator. Bioinformatics. 2021;37(8):1171–1173. 10.1093/bioinformatics/btaa783. 32926138 PMC8150139

[36] Jaganathan K, Kyriazopoulou Panagiotopoulou S, McRae JF, Predicting splicing from primary sequence with deep learning. Cell. 2019;176(3):535–548.e24. 30661751 10.1016/j.cell.2018.12.015

[37] Schatz MC, Philippakis AA, Afgan E, Inverting the model of genomics data sharing with the NHGRI Genomic Data Science Analysis, Visualization, and Informatics Lab-space. Cell Genom. 2022;2(1):100085. 35199087 10.1016/j.xgen.2021.100085 PMC8863334

